# Editorial: Microbial ecology and function of the aquatic systems

**DOI:** 10.3389/fmicb.2022.1109221

**Published:** 2023-02-15

**Authors:** Haihan Zhang, Defeng Xing, Yinhu Wu, Rencun Jin, Di Liu, Peter Deines

**Affiliations:** ^1^Shaanxi Key Laboratory of Environmental Engineering, Key Laboratory of Northwest Water Resource, Environment and Ecology, Ministry of Education, Xi'an University of Architecture and Technology, Xi'an, China; ^2^School of Environmental and Municipal Engineering, Xi'an University of Architecture and Technology, Xi'an, China; ^3^State Key Laboratory of Urban Water Resource and Environment, School of Environment, Harbin Institute of Technology, Harbin, China; ^4^Environmental Simulation and Pollution Control State Key Joint Laboratory, State Environmental Protection Key Laboratory of Microorganism Application and Risk Control (SMARC), School of Environment, Tsinghua University, Beijing, China; ^5^Laboratory of Water Pollution Remediation, School of Life and Environmental Sciences, Hangzhou Normal University, Hangzhou, China; ^6^Sandia National Laboratories, Livermore, CA, United States; ^7^Evolutionary Ecology and Genetics, Zoological Institute, Christian Albrechts University Kiel, Kiel, Germany

**Keywords:** aquatic systems, functional populations, microbial diversity, DNA-sequencing, bioturbation, biogeochemical processes

Microbes are significant elements of aquatic ecosystem as they play a critical role in the biogeochemical (C, N, P, S, and metallic element) cycling pathways, transportation of nutrients, and mitigation of pollutants (Simon et al., 2002; Sang et al., 2018; Huang et al., 2022). The community composition can vary, which gives them different ecological and environmental functions. The function of microbial communities is closely related to biogeochemical processes such as the transport of substance and energy, the degradation of organic matter, and the recycling of nutrients, which makes microbial communities essential for the maintenance of healthy aquatic ecosystems (Chen et al., 2019; Ma et al.; Zhang et al., 2023a,b). The study of active bacterial communities in aquatic ecosystems strengthens the links between environmental factors and the interactions among functional bacterial communities (Adyasari et al., 2019). Recently, molecular microbial ecology research is developing rapidly, including high-throughput sequencing technologies, multi-omics, bioinformatics and their integrated analysis (Berg et al., 2016; Yan et al., 2020; Matar et al., 2021; Huang et al., 2022).

The Frontiers Research Topic—Microbial ecology of aquatic systems—invited contributions in the following areas: (a) Relationship between water quality parameters and microbiome assemblage; (b) Functional microbial communities in different water bodies and sediments; (c) Modeling of ecosystems-based water quality data and microbiome function based multi-omic analyses; (d) Functional microbial communities in activated sludges and biofilms; (e) The relationship between microbial communities in drinking water pipelines and reactors.

This Research Topic combined the total of 5 articles to emphasis on the new findings and recent advances in various aspects of microbial ecology (i.e., functional microbial communities). The Research Topic in these papers was performed in seawater intrusion of groundwater, eutrophic water, wastewater and surface sediment in different areas of China and Ireland. Therefore, the purpose of this Editorial article is to reveal the fate of microbial communities under complex environmental and effects of microbial on biogeochemical processes are critical for improving water quality and protecting the health of aquatic ecosystems.

In this topic, Ma et al. utilized the high-throughput sequencing to clarify the microbes inhabiting the groundwater and their response to the seawater intrusion. The authors revealed that the composition and distribution of microbe in groundwater were affected by the degree of seawater intrusion and human activities. Meanwhile, the heterotrophic metabolism of microbes increased with the seawater intrusion, whereas the diversity of microbes was decreased. While the salinity of salinized groundwater was remarkably affected by the changes of tidal, the microbial community structure was not significantly affected. The results also indicated that the varying extents of seawater intrusion and the resulting environmental gradients were the primary factors affecting the diversity and structure of microbial communities in the groundwater of Beihai City.

Criado Monleon et al. investigated the distinguishing feature of the microbial communities in wastewater treatment systems and deteremined the effects on pre-treatment raw-domestic wastewater microbial communities. The authors revealed the comparative analysis of microorganisms in the on-site wastewater treatment system and pre-treatment of domestic sewage for the first time. Under the same environmental, hydrological and subsoil conditions, the analysis has been carried out in soil treatment units that has been achieved in soil clogging. The research effectively analyzed two microbial communities with different developmental states within soil treatment units at two independent study sites. This study showed that the utilization of pretreatment wastewater indicated significant changes in microbial community structure, which in turn had implications for microbial function, potential risks to public health and the environment.

Wei et al. employed the potential of *Alcanivorax* in the autotrophic carbon fixation and Fe(II)-oxidation. In this study, an *in situ* enrichment experiment was performed using a hydrothermal massive sulfide slab deployed 300 m away from the Wocan hydrothermal vent. The authors hypothesized that the characteristics and composition of microorganisms living in deep-sea hydrothermal sulfides and seawater may have fostered rich chemoautotrophic or chemoheterotrophic iron-oxidizing bacteria. The results indicated that the strain MM125-6 was capable of autotrophic carbon fixation and Fe(II) oxidization chemoautotrophically. The metabolic function of the iron-rich substrate *Alcanivorax* genus could be favorable for adaptation to various marine environments. This study was the first to perform autotrophic carbon fixation and Fe(II) oxidation from a hydrothermal vent *Alcanivorax* spp.

Wu et al. clarified the biological erosion of *Bellamya* spp. shells in the aquatic environment *via* laboratory level infection culture, validation and *in situ* microbial community structure analysis. The authors found that biocorrosives could be implanted into the CaCO_3_ layer of the shell, which created small holes in the shell, reduced the shell density, and led the shell to become fragile. The bioeroders were comprised with two phyla, namely, *Cyanobacteria* and *Proteobacteria*. Meanwhile, the interaction between *Cyanobacteria* and other bacteria promoted the biological function of “shell bioerosion”. The research identified the reason of “shell biological erosion” in aquatic environment, and provided one possible theoretical basis for the prevention and control of aquatic industry. The investigation was helpful to elucidate the cause of biological erosion of the aquaculture environment. This issue found that erosion mechanism of CaCO_3_ shells was triggered by cyanobacteria-cyanobacteria interaction and proposed a new theoretical basis.

Hou et al. evaluated organic matter degradation states and estimated bacterial community structures in sediments. The authors explained the affection of crayfish on organic matter degradation, nutrient cycling, sediment characteristics and bacterial communities. According to the functional annotation of procaryotic taxa analysis, four functional groups related to organic matter degradation were identified between the two groups with significant differences. Their findings showed that *P. clarkia* remarkably enhanced the oxidation-reduction potential values of sediments and provided favorable conditions for organic matter decomposition. In addition, *P. clarkia* also changed the composition and role of bacterial communities to increase the capacity for organic matter degradation.

## Author contributions

HZ, DX, YW, RJ, DL, and PD were topic editors for Microbial Ecology and Function of the Aquatic Systems. All authors contributed to the article and approved the submitted version.

## References

[B1] Adyasari D. Hassenruck C. Oehler T. Sabdaningsih A. Moosdorf N. (2019). Microbial community structure associated with submarine groundwater discharge in northern Java (Indonesia). Sci. Total Environ. 689, 590–601. 10.1016/j.scitotenv.2019.06.193 31279205

[B2] Berg J. S. Michellod D. Pjevac P. Martinez-Perez C. Buckner C. R. T. Hach P. F. (2016). Intensive cryptic microbial iron cycling in the low iron water column of the meromictic Lake Cadagno. Environ. Microbiol. 18, 5288–5302. 10.1111/1462-2920.13587 27768826

[B3] Chen L. Hu B. X. Dai H. Zhang X. Y. Xia C. A. Zhang J. (2019). Characterizing microbial diversity and community composition of groundwater in a salt-freshwater transition zone. Sci. Total Environ. 678, 574–584. 10.1016/j.scitotenv.2019.05.017 31078848

[B4] Huang Y. Zhang H. Liu X. Ma B. Huang T. (2022). Iron-activated carbon systems to enhance aboriginal aerobic denitrifying bacterial consortium for improved treatment of micro-polluted reservoir water: performances, mechanisms, and implications. Environ. Sci. Technol. 56, 3407–3418. 10.1021/acs.est.1c05254 35239323

[B5] Matar G. K. Ali M. Bagchi S. Nunes S. Liu W. T. Saikaly P. E. (2021). Relative importance of stochastic assembly process of membrane biofifilm increased as biofifilm aged. Front. Microbiol. 12, 708531. 10.3389/fmicb.2021.708531 34566913 PMC8461090

[B6] Sang S. L. Zhang X. Y. Dai H. Hu B. X. Ou H. Sun L. W. (2018). Diversity and predictive metabolic pathways of the prokaryotic microbial community along a groundwater salinity gradient of the Pearl River Delta, China. Sci. Rep. 8, 1–11. 10.1038/s41598-018-35350-2 30470770 PMC6251883

[B7] Simon M. Grossart H. P. Schweitzer B. Ploug H. (2002). Microbial ecology of organic aggregates in aquatic ecosystems. Aquat. Microb. Ecol. 28, 175–211. 10.3354/ame028175

[B8] Yan M. M. Chen S. N. Huang T. L. Li B. Q. Li N. Liu K. W.. (2020). Community compositions of phytoplankton and eukaryotes during the mixing periods of a drinking water reservoir: dynamics and interactions. Int. J. Environ. Res. Public Health, 17:1128. 10.3390/ijerph17041128 32053903 PMC7068298

[B9] Zhang H. Shi Y. Huang T. Zong R. Zhao Z. Ma B.. (2023a). NirS-type denitrifying bacteria in aerobic water layers of two drinking water reservoirs: insights into the abundance, community diversity and co-existence model. J. Environ. Sci (China). 124, 215–226. 10.1016/j.jes.2021.10.013 36182133

[B10] Zhang H. Yang Y. Liu X. Huang T. Ma B. Li N.. (2023b). Seasonal dynamics and co-existence patterns of phytoplankton and micro-eukaryotes in a temperate drinking water reservoir. Sci. Total Environ. 857, 159160. 10.1016/j.scitotenv.2022.159160 36195142

